# Extending Sensing Range by Physics Constraints in Multiband-Multiline Absorption Spectroscopy for Flame Measurement

**DOI:** 10.3390/s25072317

**Published:** 2025-04-05

**Authors:** Tengfei Jiao, Sheng Kou, Liuhao Ma, Kin-Pang Cheong, Wei Ren

**Affiliations:** 1 School of Aeronautics and Astronautics, Sichuan University, Chengdu 610065, China; tengfeijiao@stu.scu.edu.cn; 2 Xi’an Aerospace Propulsion Test Technology Institute, Xi’an 710100, China; 3 Combustion and Laser Sensing Laboratory, School of Automotive Engineering, Wuhan University of Technology, Wuhan 430070, China; 4 Department of Mechanical and Automation Engineering, and Shenzhen Research Institute, The Chinese University of Hong Kong, New Territories, Hong Kong SAR, China

**Keywords:** tunable diode laser absorption spectroscopy, multiband multiline absorption spectroscopy, physics constraints, wide range detection, tomographic absorption spectroscopy

## Abstract

The present numerical study proposes a technique to extend the sensing range of tunable diode laser absorption spectroscopy (TDLAS) for flame measurement by involving physics constraints on both gas condition and spectroscopic parameters in the interpretation of spectra from multiple bands. A total of 24 major spectral lines for 2 spectral segments 4029–4031 cm^−1^ and 7185–7186 cm^−1^ are determined by specially designed detection function and contribution filtering. Numerical tests on uniform and complicated combustion fields prove the high accuracy, strong robustness to noise, wide sensing range, and good compatibility with tomography. The present study provides a strong technique for future complex combustion detection with advanced laser sources of broad spectrum.

## 1. Introduction

Laser absorption spectroscopy (LAS), as a non-intrusive, spatiotemporally resolved and highly quantitative technology, has been widely applied in combustion diagnostics for the detections of various thermochemical parameters [1,2]. With the development of LAS, new applications have been extended to harsh and extreme environments of high non-uniformities [3] in recent decades, such as thermal power plants [4] and aviation propulsion systems [5,6], to facilitate data acquisition and enhance our understanding of these processes.

The two-line technique (TLT), which determines gas temperature and volume fraction based on the ratio of the integrated absorbances of two spectral lines, is widely employed due to its simplicity in both principle and experimental setup [2] over the years. However, TLT usually fails to respond precisely for the entire combustion field due to either low signal-to-ratio (SNR)-integrated absorbance or low-temperature sensitivity at a certain temperature range [7]. Theoretically, TLT [8] allows only two spectrally isolated lines to participate in the detection, leading to the line selection limits and compatibility issues with advanced, wide-range laser sources such as frequency comb [9] and the latest DFB lasers with a wide tuning range. Moreover, to apply TLT, the absorbances should be integrated over the spectral dimension; information encompassed within the spectra of transitions is compressed and even lost. Consequently, the multifold knowledge of line shape, line shifting, and others in the measured spectra of multiple absorption lines cannot be fully interpreted and utilized, and the ability and robustness in the measurement of TLT is thus limited. Note that TLT is reliable when the temperature sensitivity and SNR of the absorption signals are sufficiently high. Therefore, it is not surprising that flame measurements with TLT are reported for only limited ranges of temperature (1000–2000 K) and species concentrations [7,10,11,12].

As reported in the literature, compared to TLT, there are several ways to extend the detection range by involving more spectral information. A simple but effective way is to include the integrated absorbances (*A*_int_) of multiple lines. Karpf and Rao [13] achieved a real-time, room-temperature NO_2_ measurement with a sensitivity of sub-ppb levels by integrating the absorbance over multiple transitions, in which the overall integration is found approximately proportional to the gas concentration. Based on *A*_int_ of multilines measured by hyperspectral tomography, Ma et al. [5] successfully reconstructed the 2D thermal fields of a jet engine in 50 kHz. Later, Ma et al. [14] from the Chinese University of Hong Kong considered the line-of-sight (LOS) integrated absorbances of six absorption lines centered near 1343 nm, 2482 nm and 4183 nm to resolve the non-uniform distributions of flame temperature and volume fractions of H_2_O and CO_2_ with presumed Boltzmann profiles. This method provides the spatial distribution functions that cover the minimum and maximum values of flame temperature (*T*) and gas volume fraction (*X*). However, the presumed profiles must be known *a priori* and might not be available for turbulent flames, and the solving process is quite computationally costly. Therefore, hybrid constraints and time-averaging were later introduced [15,16] to increase the iteration speed and accuracy, and the complicated profiles in turbulent flame are simplified. On the other hand, recasting the Beer–Lambert law leads to a linear relationship between the integrated absorbance and the lower state energy; the information of multilines can be used for the linear regression of Boltzmann distribution to solve *T* and *X* [17,18]. Although, with successful demonstrations, these methods do not realize full utilization of the spectra information due to the operation of absorbance integration and might be problematic when extra gas properties besides *T* and *X* are required. Additionally, the selected absorption peaks usually consist of multiple lines, but these lines are usually combined as one, and the corresponding spectral parameters must be recalculated based on spectroscopy databases, e.g., HITRAN [19] and HITEMP [20].

An alternative way to further utilize the absorption spectra is to set the absorbance instead of their integrations as the target function [21] when quantifying gaseous parameters. In this way, additional information of multiple lines such as line shape and line shifting can be inferred, and *T*, *X*, and even more parameters are thus obtained. This idea is instinctive and straightforward. It has been demonstrated and applied in quite a few studies including room-temperature gas sensing [21,22] and non-uniform flame measurements [23]. In these studies, a spectral segment that covers several absorption features is measured, which consists of multiple absorption lines. Unfortunately, the detection ranges of *T* and *X* are still limited since high fidelity spectra are unavailable at the edge of the flames (*T* < 1200 K, *X* < 0.02) [23]. This issue mainly originates from the similarity in temperature dependencies of line strengths for significant lines at the same spectral band, which means the spectral lines only respond effectively in a similar yet limited range of temperature. With numerical demonstration, Cheong et al. [24] proposed a multiband-multiline absorption spectroscopy (MMAS) scheme to handle this problem by taking the advantages of both near-infrared (NIR) mid-infrared (MIR) lines for their strong absorption at medium to room temperature and at high temperature, respectively. However, it must be pointed out that, as multiple spectral parameters and gas properties are solved simultaneously, the overfitting or ill-posed problem arises. These problems are particularly seen in combustion scenarios, in which the spectra are affected by multicomponent collisions and measurement errors. It is also found that the solutions from the multiline method significantly depend on the completeness of the spectral description and the accuracy of the spectroscopic data. Recalibration of the spectroscopic data over wide ranges of combustion conditions might be necessary for precise measurement [23].

Hence, there are still questions that remain unsolved in MMAS: (1) Are there any physical relations or constraints to alleviate the ill-posed problem in the inversion of *T*, *X*, or other gas properties from the spectra? (2) How to select the spectral lines from various spectral bands properly? (3) Is MMAS compatible with tomography of higher dimensions, and what is its performance?

To answer these questions with solid evidence, in this study, we propose a framework of physics-constrained MMAS and demonstrate the framework with numerical simulation in zero- to two-dimensional cases. Specifically, H_2_O absorption lines are selected for the demonstration since water vapor is the main product of either hydrocarbon or zero-carbon fuel, and there are abundant lines for H_2_O at different bands with mature availability of commercial laser sources. The selections of spectral bands and lines for the present MMAS are determined based on the numerical simulation in homogeneous zero-dimensional cases with the considerations of accuracy, robustness, optical realization, and computational cost. Then MMAS and TLT are compared in the tomography imaging of both axisymmetric flames for demonstration. Further application to turbulent flames is also provided to evaluate the performance of the present MMAS in complicated cases.

## 2. Methods

### 2.1. Laser Absorption Spectroscopy

The attenuation of light intensity as the laser beam at wavenumber *ν* passing through the gaseous medium can be expressed as [8]

(1) $$\alpha {\left(\nu \right)}_{i}=P\int_{0}^{L}S_{i}\left(T\left(x\right)\right)X_{\mathrm{abs}}\left(x\right)\phi \left(\nu \right)dx$$

where *α*(*ν*)*_i_* is the spectral absorptivity or absorbance at *ν* for the *i*th spectral line, *L* is the optical path length between the laser source and receiver, *P* is the gas pressure, *X*_abs_(*x*) is the local volume fraction of absorbing species, *S_i_*(*T*(*x*)) is the line strength which depends on temperature *T*(*x*), and *ϕ*(*ν*) is the normalized line shape function whose integration over *ν* is unity. Therefore, the integrated absorbance *A_i_* can be obtained as

(2) $$A_{i}=P\int_{0}^{L}S_{i}\left(T\left(x\right)\right)X_{\mathrm{abs}}\left(x\right)dx$$

The temperature-dependent line strength *S_i_*(*T*) is calculated using the following detailed expression:

(3) $$\begin{array}{cc} S_{i}\left(T\right)= & S_{i}\left(T_{0}\right)\frac{Q\left(T_{0}\right)}{Q\left(T\right)}\exp\left[\frac{hcE_{i}^{\prime \prime }}{k}\left(\frac{1}{T}-\frac{1}{T_{0}}\right)\right]\\ & \times \left[1-\exp\left(\frac{-hc\nu_{0,i}}{kT}\right)\right]{\left[1-\exp\left(\frac{-hc\nu_{0,i}}{kT_{0}}\right)\right]}^{-1} \end{array}$$

where *Q* is the partition function, *T*_0_ = 296 K is the reference temperature, *h* is the Planck’s constant, *c* is the speed of light, *k* is the Boltzmann’s constant, and *ν*_0,*i*_ and $E_{i}^{\prime \prime }$ are, respectively, the line center and lower state energy of the *i*th transition. As the intrinsic spectroscopic properties, *ν*_0,*i*_ and $E_{i}^{\prime \prime }$ determine the unique line strength of each spectral line at different temperatures. Meanwhile, the line shape function *ϕ*(*ν*) can be affected by line shifting and broadening mechanisms simultaneously [8,25]. In most practical scenarios, *ϕ*(*ν*) is mainly determined by collisional and Doppler broadening when *ν*_0,*i*_ is known for the atmosphere pressure flame [25,26,27,28], which could be described by the Voigt profile from the convolution of collisional and Doppler broadening [29]. The effects of these broadenings can be quantified using their full widths at half maximum, Δ*ν*_*C*,*i*_ and Δ*ν*_*D*,*i*_, respectively:

(4) $$\Delta \nu_{C,i}=2P\sum_{j}X_{j}\gamma_{j}\left(T_{0}\right){\left(T_{0}/T\right)}^{n}$$

(5) $$\Delta \nu_{D,i}=\nu_{0,i}\left(7.1623\times 10^{-7}\right){\left(T/MW\right)}^{1/2}$$

where *X_j_* is the volume fraction of collisional partner *j*, *γ_j_* (*T*) is the collisional broadening coefficients at temperature *T*, *n* is the temperature coefficient which is typically 0.5–0.8, and *MW* is the molecular weight of target species. These equations, with corresponding spectroscopic parameters and the Voigt line shape function [8], together construct the basic model for the calculation of the absorption spectra.

**Figure 1:**
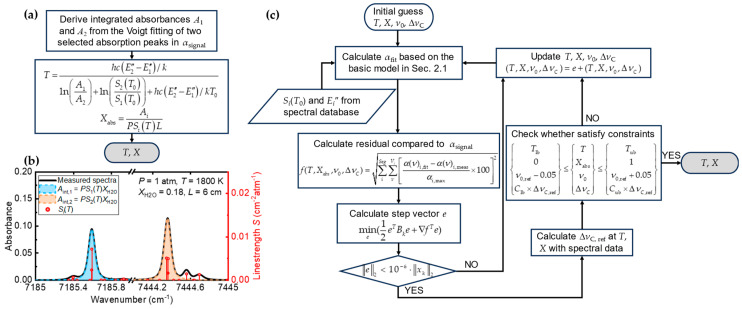
Illustrations of TLT and MMAS algorithms: (**a**) TLT, (**b**) integrated absorbances for TLT, (**c**) MMAS.

To interpret the gas parameters such as *T* and *X*_abs_ from the measured spectra, as shown in Figure 1a, TLT derives the integrated absorbance *A*_int_ (the shadow areas in Figure 1b) based on Voigt function fitting, then the ratio of *A*_int_ along with the reference spectral data of the selected two lines are applied to solve *T* and *X*:

(6) $$T=\frac{hc\left(E_{2}^{\prime \prime }-E_{1}^{\prime \prime }\right)/k}{\ln\left(A_{1}/A_{2}\right)+\ln\left(S_{2}\left(T_{0}\right)/S_{1}\left(T_{0}\right)\right)+hc\left(E_{2}^{\prime \prime }-E_{1}^{\prime \prime }\right)/kT_{0}}$$

(7) $$X_{\mathrm{abs}}=\frac{A_{i}}{PS_{i}\left(T\right)L}$$

The simplicity and fast calculation characteristics of TLT have made it popular in TDLAS measurements. However, the information about line shape and line shifting is compressed during post-processing. Hence, instead of integrating the spectra for solving gaseous parameters, we propose a method of MMAS with physics constraints and full utilization of the information contained in multiple segments of spectra from various bands. The idea and procedures are noted as follows. As seen from Equation (1), *α*(*ν*) is determined by the product of *P*, *X*_abs_, *L*, *S_i_*(*T*), and *ϕ*(*ν*). In practical measurements, *P* is commonly treated as a constant and can be further treated as atmospheric pressure under open-space conditions, while *L* can be obtained from the optical setup. Hence, the other three variables, *X*_abs_, *S_i_*(*T*), and *ϕ*(*ν*), mainly dominate the shape of the summed spectra. According to Equation (3), *S_i_*(*T*) is dependent on *T* and *ν*_0,*i*_, and *S_i_*(*T*_0_) and *E_i_* can be found in the spectroscopic database, while *ϕ_ν_* is determined by both the line-shifting mechanism and line-broadening mechanism (Δ*ν_C_*_,*i*_ and Δ*ν_D_*_,*i*_). Theoretically, as seen in Equation (5), Δ*ν_D_*_,*i*_ could be described by *T* and *ν*_0,*i*_ just like *S_i_*(*T*), but it is unlikely to obtain Δ*ν_C_*_,*i*_ without an accurate estimation of the broadening coefficients *γ_i_*(*T*), which are hard to determine due to various collisional partners in practical combustion scenarios. Therefore, under constant pressure condition, Δ*ν_C_*_,*i*_ should be considered as an implicit part, and the final spectra can be described with only four parameters: *T*, *X_abs_*, *ν*_0,*i*_, and Δ*ν_C_*_,*i*_.

According to the above discussion, the gas parameters *T* and *X*_abs_ could be theoretically solved from the spectra fitting with constraints to alleviate the multi-solution or non-physical solution issues as follows:

(8) $$\begin{array}{c} \min f(T,X_{\mathrm{abs}},v_{0},\Delta v_{C})=\sqrt{{\sum_{i}^{Seg}\sum_{\nu }^{V_{i}}\left[\frac{\alpha {\left(\nu \right)}_{i,\mathrm{fit}}-\alpha {\left(\nu \right)}_{i,\mathrm{meas}}}{\alpha_{i,\max}}\times 100\right]}^{2}}\\ \mathrm{subject}\;\mathrm{to}\left\{\begin{array}{c} T_{\mathrm{lb}}\\ 0\\ \nu_{0,\mathrm{ref}}-0.05\\ C_{\mathrm{lb}}\times \Delta \nu_{C,\mathrm{ref}} \end{array}\right\}\leq \left\{\begin{array}{c} T\\ X_{\mathrm{abs}}\\ \nu_{0}\\ \Delta \nu_{C} \end{array}\right\}\leq \left\{\begin{array}{c} T_{\mathrm{ub}}\\ 1\\ \nu_{0,\mathrm{ref}}+0.05\\ C_{\mathrm{ub}}\times \Delta \nu_{C,\mathrm{ref}} \end{array}\right\} \end{array}$$

where *a*(*ν*)*_i_*_,fit_ and *a*(*ν*)*_i_*_,meas_ are, respectively, the fitted and measured absorbance at *ν* of the *i*th spectral segment, *Seg* is the number of spectral segments, *V_i_* is the number of discrete wavenumber in the *i*th spectral segment, and *a_i_*_,max_ is the maximum value of the *i*th measured spectra to normalize the residual. As these four parameters are derived by minimizing the target function of Equation (8), multiple or non-physical (e.g., negative collisional/Doppler broadening) solutions might arise. Hence, constraints for the four unknowns are applied to avoid non-physical solutions or the unpreferred local minimum. Note that the constraints are relatively loose. For *T* and *X*_abs_, the constraints are set as their possible lowest and highest values in common flame measurements, i.e., *T*_lb_ (300 K) ≤ *T* ≤ *T*_ub_ (2500 K) and 0 ≤ *X*_abs_ ≤ 1. For *ν*_0,*i*_, it was found that the average value of the collisional shift for near-infrared H_2_O transition was around −0.017 cm^−1^/atm [8], and the Doppler shift was negligible in incompressible, open-spaced combustion; thus, ±0.05 cm^−1^ was set to cover the variations in line shifting. On the other hand, the bounds for Δ*ν_C_*_,*i*_ were adaptively set based on a dynamic reference Δ*ν_C_*_,ref_, calculated using Equation (4) with HITRAN data at the updates of *T* and *X*_abs_ during spectra fitting. This reference value was multiplied by factors of *C*_lb_ = 0.1 and *C*_ub_ = 1.9 for the evaluations of lower and upper bounds, respectively. It is worth noting that such bounds are updated according to the physical model of the spectrum; thus, the solving of the unknowns is also physically constrained by the spectral model (Equations (1)–(5) and the reference spectral data). To incorporate constraints and speed up the iteration, sequential quadratic programming (SQP) [30] is selected as the solving algorithm for Equation (8), so that the optimization is decomposed into simple quadratic programming problem by Taylor expansion:

(9) $$f(x)\approx f(x_{k})+\nabla f{\left(x_{k}\right)}^{T}e+\frac{1}{2}e^{T}B_{k}e$$

where *x_k_* represents the solution for unknows at the *k*th step, *e* is the step vector representing *x*_*k*+1_ − *x_k_*, and ∇*f*(*x_k_*) and *B_k_* are the gradient and Hessian matrix for the target function, respectively, which are determined by finite difference and quasi-Newton methods in this study. Consequently, the fitting problem in Equation (8) is transformed into minimization of variation:

(10) $$\underset{e}{\min}(\nabla f^{T}e+\frac{1}{2}e^{T}B_{k}e)$$

The flow chart for the present MMAS algorithm is illustrated in Figure 1c, which can be realized by *fmincon* of MATLAB 2016a or *SciPy* [31] based on Python 3.12.2.

### 2.2. Line Selection Criterion

As spectral transitions are only significant at certain temperature or pressure conditions, the total spectra consist of various transitions for different positions in the combustion field. Mathematically, the selection of spectral segments results in different problems of optimization formed as Equation (8). Careful selection of spectral lines or segments is critical for effective spectral information measurement and extraction. According to our previous work [7], the mid-infrared (MIR) line pair (4029.524 cm^−1^ and 4030.729 cm^−1^) and near-infrared (NIR) line pair (7185.574 cm^−1^ and 7444.364 cm^−1^) are, respectively, suitable for TLT detections of high temperature (1000 K to 3000 K) and low temperature (300 K to 1000 K). Related spectroscopic parameters are shown in Table 1. The selection criterion is based on spectral isolation, absorbance level, and temperature sensitivity.

In the present physics-constrained MMAS, the explicit constraints of each unknown are set in Equation (8), while the self-constraint by the spectral model is accomplished by spectral line selection. Line selection is a key step for MMAS, particularly in the utilization of spectral information and the robustness of problem solving for all conditions. The issue of the multi-solution is inevitable for Equation (8). However, proper selection of spectral lines or spectral segments could effectively alleviate this issue. The line selection for the present physics-constrained MMAS is conducted based on three considerations: (1) absorbance level should be above 0.01 in the main region of interest, which guarantees acceptable signal-to-noise ratio and effective measurement in the experiment; (2) high temperature sensitivity, which is proportional to the gradient of line strength; and (3) spectral isolations of main absorption lines. The first two considerations can be mathematically combined into a product term, which is detailed in Section 3.1. The third consideration is that, unlike TLT, the present method does not require an isolated main absorbance peak. Instead, it only requires that the major spectral lines are separated from each other, with a spectral distance larger than 0.001 cm^−1^. Otherwise, neither the bounds of constraints nor the self-constraint by the spectral model will work to avoid the multiple solution issue. In Section 3.1, we conduct the analysis of line selection for the proposed MMAS in detail from the above three aspects.

### 2.3. Tomography

**Figure 2:**
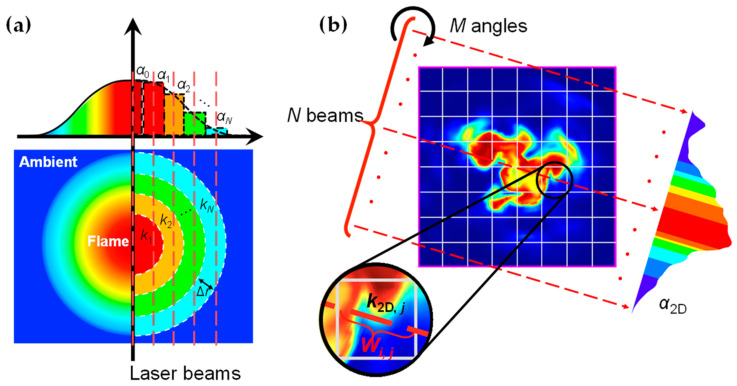
Illustration of tomography algorithms: (**a**) 1D tomography for axisymmetric flame; (**b**) 2D tomography for non-axisymmetric flame.

In practical flame measurement, tomography algorithms are necessary to reconstruct local information from projection signals depending on the symmetry of the combustion field. For axisymmetric flames, such as laminar flames from circular nozzles, one-dimensional tomography is preferred, as shown in Figure 2a. The local spectral information could be reconstructed with Abel transform:

(11) $$k\left(r\right)=-\frac{1}{\pi }\int_{r}^{R}\frac{\alpha^{\prime }\left(x\right)}{\sqrt{x^{2}-r^{2}}}dx$$

where *r* is the radial position, *α*(*x*) represents the absorbance for MMAS while it represents the integrated absorbance for TLT, and *k* is absorption coefficient for MMAS but integrated absorption coefficient for TLT. Since the LOS *α*(*x*) is usually measured evenly, Equation (11) can be transferred to a discrete form as an algebraic equation using a three-point Abel inversion scheme (ATP) [32], and Tikhonov regularization is applied to remedy its ill-posed nature:

(12) $$\left[\begin{array}{c} A_{ATP}\\ \lambda_{1D}L_{1D} \end{array}\right]k=\left[\begin{array}{c} a\\ 0 \end{array}\right]$$

where *a* = [*a*_1_, *a*_2_, …, *a_N_*]^T^ is the discrete projection value, *k* = [*k*_1_, *k*_2_, …, *k_N_*]^T^ is the radially reconstructed local value, *A_ATP_* is the inverse matrix of ATP deconvolution operator (*D_ATP_*) [7], and λ_1D_ is the Tikhonov regularization parameter for 1D tomography determined using the L-curve method [33]. *L*_1D_ is the 1D tomography smoothing matrix:

(13) $$L_{1D,ij}=\left\{\begin{array}{c} 1,\;\mathrm{if}\;i=j\\ -1,\;\mathrm{if}\;i=j+1\\ 0,\;\mathrm{otherwise} \end{array}\right.$$

More details about 1D tomographic absorption spectroscopy can be found in the literature [34]. On the other hand, for non-axisymmetric flames, such as turbulent flames, multiple measurements from different positions and directions are required rather than one angle, as shown in Figure 2b. The reconstruction of the local spectra in this work results in a large-scale matrix. Therefore, a simultaneous algebraic reconstruction technique (SART) [3,35,36] with GPU acceleration [37,38] is applied due to its lower computation time and compatibility with parallel computing:

(14) $$k_{j}^{n+1}=k_{j}^{n}+\beta \frac{\sum_{i=1}^{N\times M}\frac{a_{i}-\sum_{j=1}^{J}W_{ij}k_{j}^{n}}{\sum_{j=1}^{J}W_{ij}}W_{ij}}{\sum_{i=1}^{N\times M}W_{ij}}$$

where *W_ij_* is the weight matrix representing the optical length of the *i*th beam in the *j*th pixel, $k_{j}^{n}$ is the iterative solution at the *n*th iteration, *β* = 1 is the relaxation factor controlling the rate of convergence in this work, *a_i_* is the projection signal for the *i*th beam, *M* is the total number of angles, *N* is the number of parallel beams, and *J* is the total pixel number.

## 3. Results and Analysis

### 3.1. Selection and Validation of Spectral Lines in Uniform Thermal Field

Common spectral segments selected in TDLAS measurements for H_2_O in previous studies spread from NIR to MIR, as shown in Table 2. Considering the commercial availability of laser sources, four spectral segments of 4029–4031 cm^−1^, 7185–7186 cm^−1^, 7306–7307.5 cm^−1^, and 7444–7445 cm^−1^ are selected as candidates. These four segments are, respectively, abbreviated as Segment 4029, 7185, 7306, and 7444.

**Figure 3:**
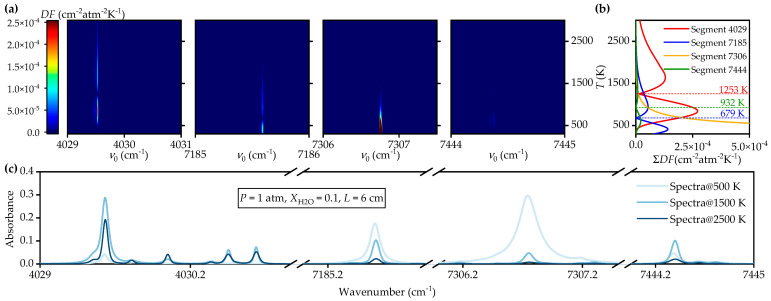
(**a**) Detection function *DF* of 4 segments; (**b**) Segmental summation of DF and the cusp points; (**c**) Simulated spectra at 500 K, 1500 K and 2500 K with *P* = 1 atm, $X_{H_{2}O}$ = 0.1 and *L* = 6 cm.

As mentioned in Section 2.2, line selection for MMAS should satisfy the requirements of absorbance level, temperature sensitivity, and spectral isolation. To evaluate these factors for the candidate spectral segments quantitatively, particularly in terms of absorbance level and temperature sensitivity, a detection function *DF* is designed in this section:

(15) $$DF=\left|\left[\phi \left(\nu_{0}\right)\cdot S\left(T\right)\right]\frac{d\left[\phi \left(\nu_{0}\right)\cdot S\left(T\right)\right]}{dT}\right|$$

where *A*_p_(*ν*_0_) = *ϕ*(*ν*_0_)·*S*(*T*) is the nominal peak absorption value of each transition and can be regarded as the absorption value at the line center with *P* = 1 atm, $X_{H_{2}O}$ = 1, and *L* = 1 cm, respectively. With *DF*, the information about line strength and line shape and their sensitivity to temperature are simultaneously analyzed. A large *DF* of a specific absorption line represents its good signal-to-noise ratio (SNR) or high-temperature sensitivity in the measurement. During the stage of line selection, *A*_p_(*ν*_0_) is calculated with the HITRAN database [19]. The values of *DF* against *T* for various absorption lines within the candidate spectral segments are displayed with color scale in Figure 3a, along with their segmental summation plotted in Figure 3b and typical spectra in Figure 3c.

Here, we discuss the spectral line or segment selection rules for the physics-constrained MMAS. The *DF* displayed in Figure 3a indicates that all the candidate segments are only responsive or sensitive within the limit temperature range. For example, Segment 4029 is sensitive mainly based on the information provided by the lines near 4029.7 cm^−1^. However, this segment might not work well when the temperature is less than 500 K or around 1250 K, as the values of *DF* are small for these temperatures. Similar phenomena can be observed for other three segments with different sensitive ranges of temperature. Compared to the *DF* in other segments, the *DF* of Segment 7444 is much smaller so that the spectral information provided by this segment is not so effective. To illustrate more clearly, we summarized all the *DF*s of different lines within a segment, as shown in Figure 3b. It clearly and quantitatively demonstrated the observations from Figure 3a. Equally important, it is shown that there are two cusp points for the *DF* of Segment 4029, and the *DF* of Segment 7185 is a good compensation around 300 K and 1253 K. Although Segment 7306 is very sensitive at room and immediate temperatures, it is less responsive when *T* > 1000 K, and Segment 7444 is not ideal for MMAS. Therefore, the combination of Segments 4029 and 7185 provides effective information from low to high temperatures. The typical spectra in Figure 3c show that these two segments provide useful detections for the target temperatures.

On the other hand, as we can see from Figure 3a, the spectral information is provided by only a few lines. Therefore, we perform spectral line filtering to reduce the number of lines so that the calculation can be accelerated. The line reduction is conducted by considering the parameter ranges of *T* ∈ [300 K, 2500 K], $X_{H_{2}O}\in$ [0.02, 0.2], and *C*_M_ ∈ [0.1, 1.9] where *C*_M_ is a random coefficient accounting for the uncertainties in the air collisional broadening coefficient, $\gamma_{H_{2}O-\mathrm{air}}$, with its relation to collisional broadening as

(16) $$\Delta \nu_{C}=2PX_{H_{2}O}\gamma_{H_{2}O-H_{2}O}+2P\left(1-X_{H_{2}O}\right)\gamma_{H_{2}O-\mathrm{air}}\times C_{M}$$

By calculating the absorbance with different parameters, the contributions of the spectral lines are sorted in descending order. Those lines, with a total contribution less than 1%, are ignored. The filtered major lines for the two candidate segments are shown in Table 3. As a result, 24 spectral lines are filtered from 275 lines documented in HITRAN.

**Figure 4:**
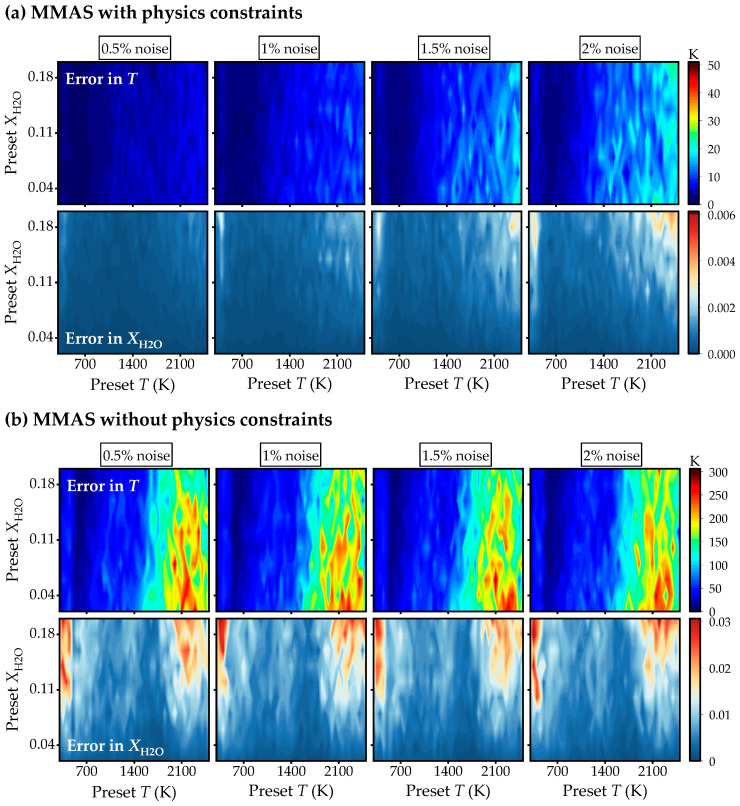
Mean absolute error in recovered *T* (**top**) and $X_{H_{2}O}$ (**bottom**) in uniform fields with different noise levels: 0.5%, 1%, 1.5%, and 2%: (**a**) with physics constraints; (**b**) without physics constraints.

After line selection, numerical verification in a uniform field is conducted to evaluate the robustness and accuracy of the selected segments under various measurement scenarios, including variations in gas parameters, noise levels, and molecular collisions. Note that the simulated spectra apply detailed line parameters from HITRAN with random coefficient *C*_M_ and noise levels; then these spectra are fitted and interpreted by present MMAS with the filtered spectral lines displayed in Table 3. The random noises are generated using Gaussian noise. All verification is repeated 100 times under each noise level with mean absolute error shown as contours in Figure 4. All the simulations are performed on a workstation with two Intel(R) Xeon(R) Platinum 8275CL CPUs and 48 GB RAM.

As illustrated in Figure 4a, the maximum errors of the interpreted *T* and $X_{H_{2}O}$ by the present MMAS are, respectively, less than 30 K and 0.004, even in high-temperature conditions, which is quite satisfying. The high errors of *T* and $X_{H_{2}O}$ mainly concentrate in the regions with preset *T* > 1500 K or $X_{H_{2}O}$ > 0.15, likely the highly reactive zone in the combustion field. This is also expected from Figure 3b, since *DF* decreases at a high temperature. On the other hand, the high fraction of target species increases uncertainties in the estimation of collisional broadening. To demonstrate our idea more clearly, we have evaluated the errors in *T* and *X* by using MMAS without physical constraints, as shown in Figure 4b. The errors significantly increase up to 300 K and 0.03, respectively, which may worsen if the noise level increases. This is mainly due to the reason that, without the constraints, MMAS cannot effectively handle the multisolution issues, because it derives the solution mathematically optimal (small deviations in spectra fitting) but not physically reasonable (significant errors in *T*, *X_abs_*, *ν*_0,*I*_ and *Δν_C_*_,*i*_). It is, thus, confirmed that the solving algorithm with filtered spectral lines offers accuracy and robustness to noise and variations of collisional parameters. In practice, the tomography algorithm introduces additional errors in the spectra. Therefore, applications of the present MMAS method to axisymmetric and two-dimensional combustion fields are conducted in order to evaluate the performance of the present method in real-flame measurements. Traditional TLT is served as a benchmark in the following sections.

### 3.2. Axisymmetric Flame

**Figure 5:**
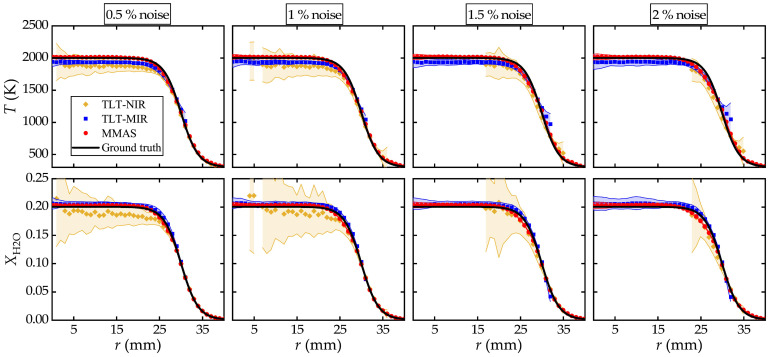
Reconstructed profiles of temperature (**top**) and $X_{H_{2}O}$ (**bottom**) from MMAS, TLT-MIR and TLT-NIR for axisymmetric flame with different noise levels.

In this test, to generate an axisymmetric flame, the distributions of temperature and H_2_O fraction are set following the flat flame with Boltzmann distribution proposed by Ma et al. [14]:

(17) $$F(r)=\frac{a_{1}-a_{2}}{1+e^{\frac{r-a_{3}}{a_{4}}}}+a_{2}$$

where *a*_1_ = 2000, *a*_2_ = 300, *a*_3_ = 30, *a*_4_ = 2 for radial temperature *T*(*r*), and *a*_1_ = 0.2, *a*_2_ = 0, *a*_3_ = 30, *a*_4_ = 2 for radial H_2_O concentration $X_{H_{2}O}$(*r*). The spatial resolution (Δ*r*) is 1 mm. For MMAS, information from Segments 4029 and 7185 is utilized, while the two-line pairs shown in Table 1 are applied in TLT and noted as TLT-MIR and TLT-NIR, respectively. The numerical test is similar to those in Section 3.1 at different noise levels, 0.5%, 1%, 1.5%, and 2%, except that the local spectra have to be reconstructed using Abel inversion. The random test is repeated 100 times to gain the standard deviation as uncertainty, with results illustrated in Figure 5.

As seen in Figure 5, neither TLT-NIR nor TLT-MIR reconstructs the full radial profiles. TLT-MIR fails to work when the temperature is lower than 1000 K due to the significant uncertainty in extracting the integrated absorbance from the spectra of low absorbance, while TLT-NIR deviates from the ground truth when the temperature is above 1500 K, although it detects quite well under low temperature. In contrast, MMAS reconstructs the full distributions of temperature and $X_{H_{2}O}$ despite the increment in noise levels. These are facilitated by the multiple information and constraints provided by the multiple lines in the two spectral segments that are sensitive to the whole ranges of the parameters.

### 3.3. Two-Dimensional Combustion Field

Turbulent flames appear more frequently in our daily life and industrial processes, such as those in thermal power plant or aerospace propulsion system. The unsteady and random features of turbulence results in the failure of Abel inversion for axisymmetric reconstruction. Therefore, the 2D tomography algorithm is introduced as shown in Figure 2b. The ground truths of temperature and H_2_O distributions are extracted from computational fluid dynamic (CFD) simulation of turbulent jet flame based on large eddy simulation (LES) framework of Wang et al. [48]. The CFD results are discretized into pixels of 25 × 25 with a spatial resolution of 7.2 mm. A scheme of parallel beams with beam number of 25 and angular number of 20 after parametric study, in which the computational cost and precision are balanced. During reconstruction, double masking operation is proposed to alleviate the effect of artifacts. Specifically, the first masking operation is to eliminate inactive pixels by setting threshold ($X_{H_{2}O}$ = 0.001) to the projection values so that only the effective pixels with sufficient contribution to the projections are participated in the reconstruction [47,49,50]. The second masking operation is to distinguish the edge of the flame after the reconstruction with the same threshold ($X_{H_{2}O}$ = 0.001). The reconstruction error is statistically evaluated from multiple comparisons between the reconstructed and the CFD results following:

(18) $$Error=\frac{1}{N_{v}}\sum_{i}^{N_{v}}\left|V_{RE}-V_{GT}\right|$$

where *V_RE_* means the reconstructed value and *V_GT_* means the value from ground truth, *N_v_* means the number of valid pixels where reconstruction works and those are not masked.

**Figure 6:**
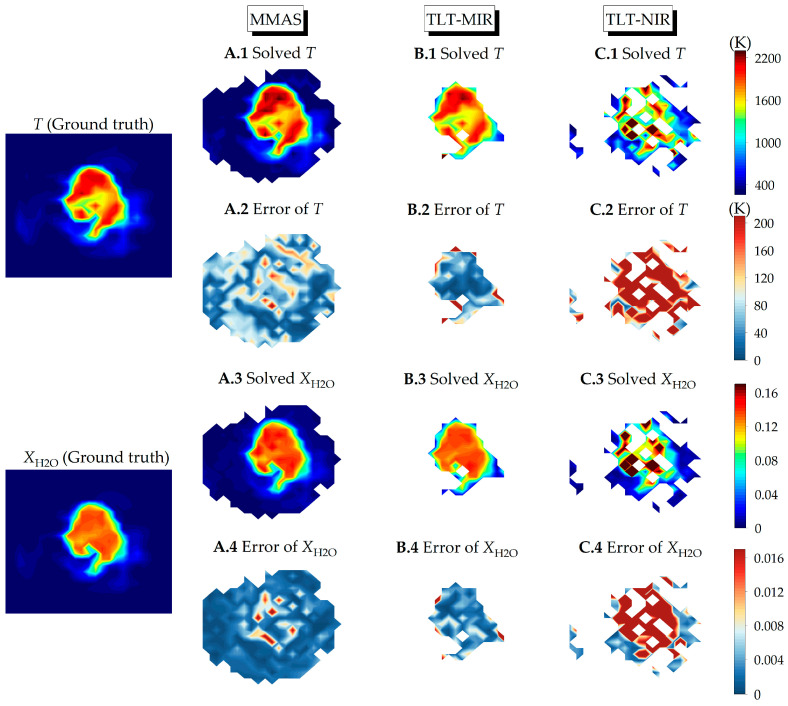
Reconstructions of *T* and $X_{H_{2}O}$ 2D distributions by (**A**) MMAS, (**B**) TLT-MIR and (**C**) TLT-NIR with 0.5% Gaussian white noise. The leftmost part is the reference *T* (**top**) and $X_{H_{2}O}$ (**bottom**).

Figure 6 presents the typical reconstruction results from MMAS and TLT with a noise level of 0.5%. Note that only the pixels with valid results and not being masked are displayed. Similar to the axisymmetric cases, TLT-MIR performs satisfyingly in high temperature regions but fails to work in low temperature region. Meanwhile, TLT-NIR deviates significantly from the ground truth or even fails at high temperature region. On the other hand, MMAS realizes effective detection for most of the turbulent flame with satisfying accuracy. It is worth noting that at high temperature region, MMAS might be less accurate than TLT-MIR, which is mainly caused by the distortion of local spectra during reconstruction. Therefore, obtaining high fidelity spectra is essential for the good performance of MMAS in 2D turbulent monitoring.

**Figure 7:**
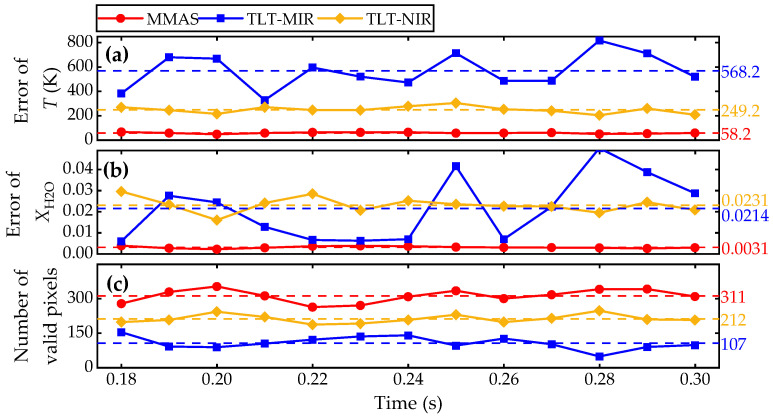
Mean reconstruction errors of MMAS and TLT in consequent 2D tomography test with 0.5% Gauss white noise.

We further perform the reconstructions of time-history 2D distributions of the combustion field to evaluate the performance of the present method. The reconstructed errors calculated using Equation (18) and numbers of valid pixels are plotted in Figure 7. Note that the time interval of CFD results is 0.01 s and all the tomographic reconstructions are conducted with a Gaussian noise level of 0.5%.

As shown in Figure 7a,b, the mean errors of *T* and *X* in the 2D tomography test for MMAS are only 1/4 or 1/10 to those of TLT-NIR and TLT-MIR, respectively, and the number of valid pixels in the reconstruction increases notably (Figure 7c). Among these methods, TLT-MIR seems to be the most unstable one, which may be caused by the least valid pixels. The low absorbance level at low temperatures for the MIR segment induces a high possibility of mismatching between the detected field and beam arrangement. Even though there are quite a lot of valid pixels in TLT-NIR, the low temperature sensitivity at the high temperature region for the NIR segment leads to a high level of mean error. For MMAS, the utilization of spectra from the multiband prevents the issues in temperature sensitivity and the number of valid pixels. From Figure 7, it is concluded that MMAS outperforms the conventional TLT algorithm at the expense of heavier computational cost or lower calculation speed.

**Figure 8:**
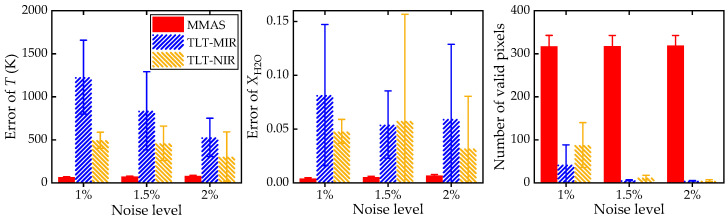
Behaviors of MMAS and TLT in consequent 2D tomography test, respectively, with 1%, 1.5%, and 2% Gauss white noise. Left: error of *T*; middle: error of $X_{H_{2}O}$; right: number of valid pixels. The error bars represent the uncertainties of these mean errors obtained from 100 random tests.

To further verify the robustness to noise of MMAS and TLT in 2D tomography, numerical tests with noise levels of 1%, 1.5%, and 2% are conducted, as illustrated in Figure 8. It is clear to see that the number of valid pixels decreases dramatically for TLT-MIR and TLT-NIR. In comparison, for MMAS, the number of valid pixels is maintained around 300, regardless of the noise level. MMAS effectively reconstructs the combustion fields with high accuracy and a widely applicable temperature range. The above verification reveals two interesting results: (1) the influence of noise in 2D tomography is much more significant than that in the 1D field, especially when the number of optical accesses is limited; (2) the present physics-constrained MMAS with SART and double-masking operation shows stronger and more stable capability in reconstructing turbulent flames than TLT.

## 4. Conclusions

To extend the sensing range of TDLAS for flame measurements, a physics-constrained MMAS is proposed and numerically validated in this work. Information from multiband, multiline spectra is interpreted to obtain flame parameters, while reducing the dependency on spectroscopic data, mitigating the multisolution issue and avoiding a non-physical solution. The gas temperature and species volume fraction, *T* and *X*, and the spectroscopic parameters of line centers and collisional broadening *ν*_0_ and Δ*ν_C_* are included as unknowns with respective constraints during the fitting of the spectra. A detection function composed of peak absorbance value and temperature sensitivity is designed for line selection. With spectral line filtering, 24 major spectral lines spreading in Segments 4029 and 7185 are determined. Numerical validation in the uniform field shows strong capability of physics-constrained MMAS on accuracy and robustness to noise and uncertainties in spectroscopic data. The evaluated errors for *T* and $X_{H_{2}O}$ are below 30 K and 0.04, respectively. Further comparison with TLT in the non-uniform field demonstrated that the present physics-constrained MMAS shows similar compatibility with tomography algorithms like TLT, with even higher accuracy, stronger robustness, and a wider sensing range.

On the other hand, it should be pointed out that, compared with TLT, the present method requires more calculation and additional laser sources. Our future work will concentrate on adapting AI or other techniques to reduce solving time and experimentally realize 2D and 3D flame tomography with multiple tunable or broadband spectrum laser sources.

## Figures and Tables

**Table 1:** Parameters for spectral lines applied in TLT.

Line Pair	Wavenumber (cm^−1^)	*S*@296K (cm^−2^ atm^−1^)	*E*″ (cm^−1^)	Δ*E*″ (cm^−1^)
MIR	4029.524	1.11 × 10^−4^	2660.95	2228.534
	4030.729	2.59 × 10^−9^	4889.488	
NIR	7185.574	1.99 × 10^−2^	1045.058	750.973
	7444.364	1.08 × 10^−3^	1796.031	

**Table 2:** Previous wavenumber segments applied for H_2_O detection.

	Candidate Spectral Segments in Wavenumber (cm^−1^)
MIR	3459.65–3460.7 [39], 3981.75–3982.95 [40], 4029.0–4031.0 [7], 7153.4–7154.6 [41]
NIR	7164.5–7166.5 [42], 7185.0–7186.0 [7,43,44], 7306–7307.5 [45,46,47], 7444.0–7445.0 [7,43,44]

**Table 3:** Filtered spectral lines in Segments 4029 and 7185.

Segment	4029			7185
Line center (cm^−1^)	4028.178363 4028.25667 4028.733351 4029.428428 4029.523702 4029.524128	4029.732972 4029.779592 4030.022402 4030.091518 4030.36021 4030.492872	4030.51014 4030.568963 4030.728405 4030.729398 4030.734884 4031.402871	7185.394189 7185.400573 7185.442365 7185.596113 7185.596433 7185.936277

## Data Availability

The data presented in this study are available on request from the corresponding author.
